# Epidemiological Profile and Spatial Patterns of Enterobiasis in Children Aged 3–9 Years in China from 2016 to 2020

**DOI:** 10.3390/tropicalmed8010025

**Published:** 2022-12-29

**Authors:** Jilei Huang, Huihui Zhu, Changhai Zhou, Tingjun Zhu, Mizhen Zhang, Yingdan Chen, Menbao Qian, Shizhu Li

**Affiliations:** 1 National Institute of Parasitic Diseases, Chinese Center for Disease Control and Prevention (Chinese Center for Tropical Diseases Research), NHC Key Laboratory of Parasite and Vector Biology, WHO Collaborating Center for Tropical Diseases, National Center for International Research on Tropical Diseases, Shanghai 200025, China; 2 Hainan Tropical Diseases Research Center (Hainan Sub-Center, Chinese Center for Tropical Diseases Research), Haikou 571199, China; 3 School of Global Health, Chinese Center for Tropical Diseases Research-Shanghai Jiao Tong University School of Medicine, Shanghai 200025, China

**Keywords:** *E. vermicularis*, enterobiasis, children, spatial and temporal distribution characteristics

## Abstract

(1) Background: *Enterobius vermicularis* infection causes a significant health burden in children. The infection occurs throughout the country and remains a serious public concern in China. Therefore, it is necessary to know the situation of *E. vermicularis* infection, to provide a scientific basis for the disease control and the optimum conditions for children’s growth. (2) Methods: Descriptive epidemiological analysis was implemented to demonstrate the status and changing trend of *E. vermicularis* infection from 2016 to 2020, while the spatial distribution characteristics and spatial-temporal clustering were illuminated by spatial autocorrelation analysis and spatio-temporal scanning analysis. (3) Results: The infection of *E. vermicularis* showed a fluctuating downward trend with a decline of 32.00% in 2020 compared to that in 2016 and was concentrated in central and southern China. There was no significant difference in infection rate between boys and girls, while the high infection rate was presented in 4-, 5- and 6- year-old children. The hotspots and spatial clustering areas were mainly concentrated in southern China. (4) Conclusions: From 2016 to 2020, the infection rate of *E. vermicularis* in children aged 3 to 9 years in China demonstrated a declining trend, and its distribution showed spatial clustering, mainly in southern China. Therefore, it is necessary to strengthen surveillance and implement control measures in combination with health education and environmental improvement.

## 1. Introduction

Enterobiasis caused by *Enterobius vermicularis* infection is widely distributed around the world [1,2]. Transmission usually occurs among children in childcare agencies and kindergartens [3,4,5]. The main symptoms of enterobiasis include perianal skin itching, night terrors, grinding of teeth, irritability, anorexia and emaciation, but it may also cause appendicitis and urogenital system inflammation due to ectopic parasitism [6,7]. A long-term repeated infection will result in delayed growth and development of children and affect their physical and mental health [8,9,10]. Enterobiasis is prevalent in China [11], in spite of rapid economic and social development, improved quality of life and significantly enhanced health knowledge and awareness. According to the survey of national key human parasitic diseases in China in 2015, the infection rate of *E. vermicularis* was 0.33%, with an estimation of 2.14 million persons under infection. A high infection rate was presented in some areas (i.e., 3.85% in Hainan Province and 1.24% in Jiangxi Province) [12].

To boost the Healthy China initiative, the Chinese government issued “The national program for prevention and control of echinococcosis and other key parasitic diseases in 2016–2020” (hereinafter referred to as "the prevention and control program") and implemented programs for the prevention and control including a nationwide surveillance network for key parasitic diseases [13,14,15]. Since 2016, enterobiasis has been included in the national surveillance. The distribution characteristics and spatio-temporal clustering of *E. vermicularis* infection in children aged 3 to 9 years old in China between 2016 and 2020 are analyzed here, to provide a scientific basis for formulating the prevention and control programs of enterobiasis in future.

## 2. Methods

### 2.1. Sampling

According to “China’s national surveillance program for clonorchiasis and soil-borne nematodiasis (Trial)” (hereinafter referred to as “the surveillance program”), counties (cities and districts) from 31 provinces (autonomous regions and municipalities) in China were taken as the units and one or two counties were selected from each province to perform fixed site monitoring. Ten to fifteen percent of the counties were then selected for mobile site monitoring, where the monitoring sites were not repeated within 5 years. A total of 1324 monitoring sites were selected in this study. Each monitoring site was divided into five areas according to its geographical location (eastern, western, southern, northern and central areas). One township (town) was randomly selected from each area, and then, one administrative village was further selected from each township. About 200 permanent residents over 3 years old were chosen from each administrative village every year. Thus, a total of 1000 persons were surveyed at each monitoring site and the average proportion of children aged 3 to 9 years was usually not less than 10%. Those children aged 3 to 9 years old were eligible for surveillance of enterobiasis.

### 2.2. Detection of Infection

One stool sample was collected from each participant, and the modified Kato–Katz thick smear method [16] was adopted to detect helminth eggs. In children aged 3 to 9 years old, the adhesive cellophane-tape anal swab method [17] was simultaneously applied to detect eggs of *E. vermicularis*. In brief, separate the anus of the child with the index finger and thumb, expand the perianal fold, then press the clear adhesive sticker repeatedly on the anal opening. The adhesive sticker is placed on the slide and then examined under microscope. If eggs of *E. vermicularis* were detected by any of the two methods, the individual was defined as positive for *E. vermicularis* infection.

### 2.3. Database and Statistical Analysis

The detection results and personal information of the subjects at each monitoring site were registered in the recording forms and entered into the surveillance system. In combination with the GPS coordinates of each monitoring site, ArcGIS 10.2 software was used to establish the spatial distribution database of *E. vermicularis* infection in children aged 3 to 9 in China from 2016 to 2020. 

Descriptive epidemiological analysis was used to analyze the epidemiological characteristics of *E. vermicularis* infection among children aged 3 to 9 years in China from 2016 to 2020. The survey data were statistically analyzed with Microsoft Excel 2016 and SAS 9.3, and *E. vermicularis* infection rate in children aged 3 to 9 years was calculated. The *χ*^2^ test was used to test significance, with a *P* value less than 0.05 indicating a statistically significant difference.

### 2.4. Spatial Autocorrelation Analysis

Global spatial autocorrelation analysis was conducted with the global spatial autocorrelation index in Moran’s *I* in the ArcGIS(Esri® 10.2, Redlands, CA, USA) software [18,19,20], and the spatial clustering of *E. vermicularis* infection in children was assessed according to the location of elements and attribute values. Hot spot analysis was carried out using Getis-Ord *G*_i_* (ArcGIS 10.1, Esri, Redlands, CA, USA) [21,22], and each element in the data set was analyzed to obtain the Z score and *P* value. These were used to determine the clustering location of the high-value (hot spot) or low-value (cold spot) elements of *E. vermicularis* infection in children with statistical significance. The hot spots with statistical significance were detected by scanning for elements with high *P* and Z values, which were surrounded by other elements with the same high values, thereby identifying high-value cluster areas of *E. vermicularis* infection in children.

### 2.5. Spatio-Temporal Analysis

SaTScan (v9.5, Boston, MA, USA) [23] software was used for retrospective spatio-temporal scanning analysis to explore cluster status. The scanning was conducted at different times and areas through scanning windows with dynamically changed sizes and positions. The theoretical number of infected people was calculated according to the number of infected people and investigated people. The actual and theoretical numbers of cases inside and outside the scanning windows were used to construct the Log likelihood ratio (LLR) of test statistics, to evaluate the abnormality of the number of infected people in the scanning window [23,24]. At the same time, the Monte Carlo method was used as the statistical test on the possible abnormal windows, and *P* < 0.05 indicated statistical significance. On the premise of statistical significance, a larger LLR value suggested a greater probability that the area under the scanning window would be a cluster area [25]. In this study, a Poisson model was adopted for the SaTScan spatio-temporal scanning analysis, and the maximum scanning window was set to 50% of all the population at risk in the study area.

## 3. Results

### 3.1. Overall Infection

**Figure 1:**
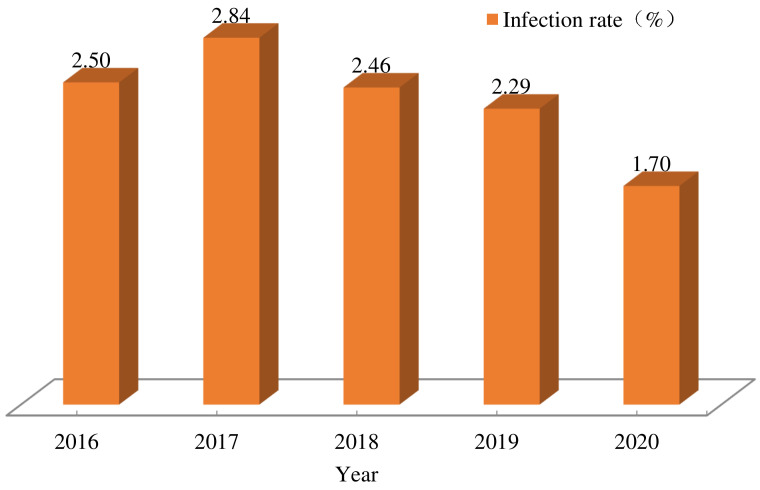
*Enterobius vermicularis* infections among children aged 3 to 9 years old in China from 2016 to 2020. The differences of the annual infection rates were statistically significant from 2016 to 2020 (*χ*^2^ = 159.02, *P* < 0.0001).

Out of 245,846 children aged 3 to 9 years in 1324 counties investigated from 2016 to 2020, 5683 ones were infected with *E. vermicularis*. The average infection rate was 2.31%, while the annual infection rates were 2.50% (911/36,477), 2.84% (1212/42,629), 2.46% (1133/46,087), 2.29% (1459/63,592) and 1.70% (968/57,061), respectively. The infection rate in 2020 decreased by 32.00% compared to that in 2016 (Figure 1 and Table 1).

### 3.2. Regional Distribution

Provinces with high *E. vermicularis* infection rate were mainly distributed throughout southern China. Those with an average infection rate above 5% during the five years included Jiangxi, Guangxi, Hainan and Fujian provinces, with an infection rate of 10.15%, 7.05%, 6.96% and 5.56%, respectively. The infection rate in 11 provinces ranged from 1% to 5%, while the remainder was less than 1% (Table 1).

Among the focus areas, the infection rate of *E. vermicularis* in children in Jiangxi decreased year by year from 2016 to 2019 but increased slightly in 2020. The infection rates of the five years were 12.12%, 14.29%, 8.52%, 6.83% and 7.81%, respectively, and the infection rate decreased by a total of 35.56% over the five years (Table 1). The infection rates in Guangdong and Guangxi decreased by 49.09% and 26.32%, respectively, over the five years. The infection rate in Hainan province fluctuated from year to year. The infection rate in Fujian firstly increased from 2016 to 2019, and then decreased in 2020 (Table 1).

### 3.3. Population Distribution

The average infection rate was 2.31% (3015/130,419) in boys and 2.31% (2668/115,427) in girls, and there existed no statistical difference in each year (*χ*^2^ = 0.29, *P* = 0.59; *χ*^2^ = 0.13, *P* = 0.72; *χ*^2^ = 0.59, *P* = 0.44; *χ*^2^ = 0.21, *P* = 0.65; *χ*^2^ = 0.07, *P* = 0.80) (Table 2). The infection rates in boys and girls decreased by 30.49% and 33.86%, respectively, over the five years.

From 2016 to 2020, the infection rate in children from Dong, Han, Hui, Miao, Tujia, Uygur, Yao and Zhuang ethnic groups decreased, while those from Buyi, Kazak and Li increased. There were significant differences in the infection rates among the ethnic groups (*χ*^2^ values of 247.19, 453.83, 361.89, 608.68 and 183.41, respectively, with *P* < 0.0001). The infection rate in children from Zhuang was the highest from 2016 to 2019 (8.35%, 12.93%, 9.63% and 9.86%). In 2020, the infection rate in children from Li was the highest (8.06%), and that from Zhuang ranked second (2.83%). The infection rate in children from Zhuang decreased by 66.11% over the five years, while that in children from Li increased by 164.26%. The average infection rate in children from Han was 2.43% (4708/193,402) and that from other ethnic groups was 1.90% (975/52,444) (*χ*^2^ = 60.44, *P* < 0.0001) (Table 2).

**Figure 2:**
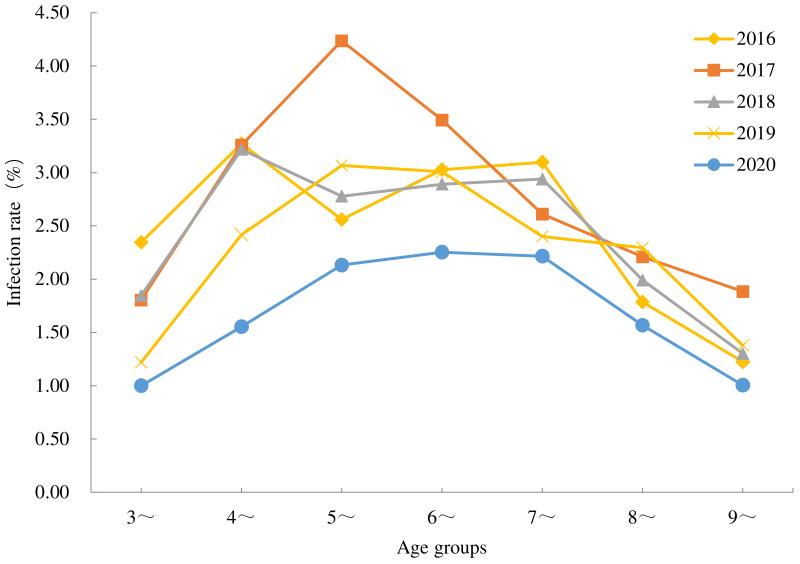
*Enterobius vermicularis* infection among children by ages in China from 2016 to 2020. The difference in infection rate was significant among ages in each year (*χ*^2^ values of 70.32, 112.62, 79.89, 128.73 and 84.33, respectively, with *P* < 0.0001).

From 2016 to 2020, the infection rate of all ages decreased, and the infection rates in the age of 3-, 4-, 5-, 6-, 7-, 8- and 9-year-old decreased by 57.45%, 52.29%, 16.80%, 25.74%, 28.39%, 12.29% and 17.89%, respectively. In 2016 and 2018, the 4-year-old group had the highest infection rate and the 3-year-old group had the lowest. In 2017 and 2019, the 5-year-old group had the highest infection rate and the 9-year-old group had the lowest. In 2020, the 6-year-old group had the highest infection rate and the 3-year-old group presented the lowest. The infection rates were significantly different by ages in each year (Figure 2; Table 3).

### 3.4. Global Spatial Autocorrelation Analysis

Moran’s *I* coefficients from 2016 to 2020 for the global spatial autocorrelation analysis at the provincial level ranged from 0.15 to 0.32, which was greater than expected. Meanwhile, the Z values were all > 0, and the significance values were all *P* < 0.01 (Table 4), suggesting that there was spatial clustering in the provincial distribution of *E. vermicularis* infection in children in China during these five years. Global spatial autocorrelation analyses at county levels revealed Moran’s *I* coefficient to be 0.08 to 0.17, also greater than expected. Z values were >0 and *P* values were 0, suggesting that the infection was spatially clustered.

### 3.5. Hotspot Analysis

**Figure 3:**
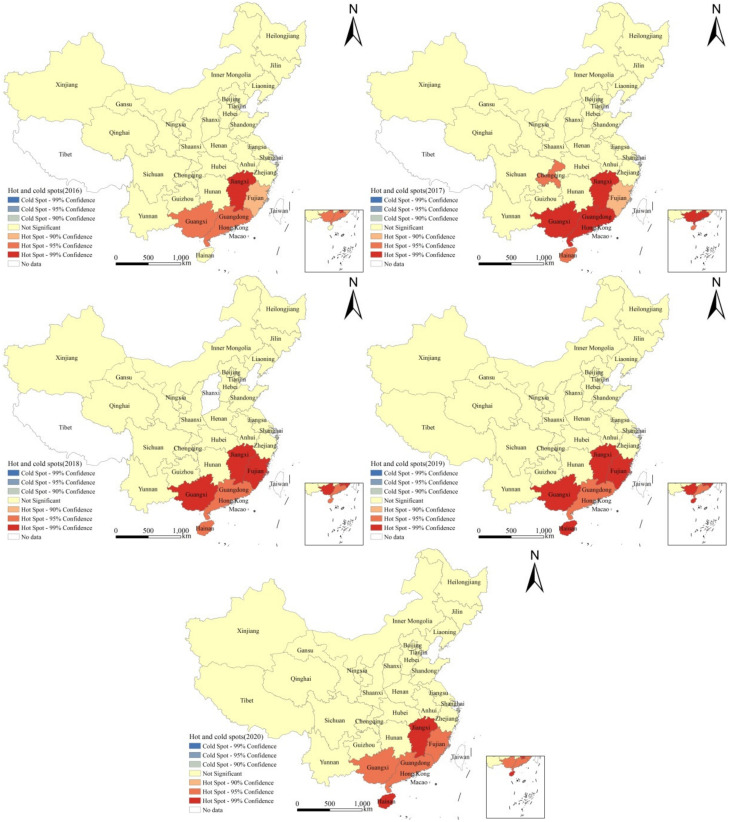
The provincial results of Getis-Ord Gi* from 2016 to 2020.

The hotspot analysis at the provincial level (Figure 3) identified four “hot spot” areas, namely Jiangxi, Guangdong, Guangxi and Fujian in 2016. In 2017, there were six “hot spot” areas, including Jiangxi, Guangdong, Guangxi, Hainan, Chongqing and Fujian. There were five “hot spot” areas in 2018, namely, Jiangxi, Guangxi, Fujian, Guangdong and Hainan, and there were five “hot spot” areas in 2019, including Jiangxi, Guangxi, Fujian, Hainan and Guangdong. There were five “hot spot” areas in 2020, namely, Jiangxi, Hainan, Guangdong, Guangxi and Fujian. There were no “cold spot” areas during the five years.

**Figure 4:**
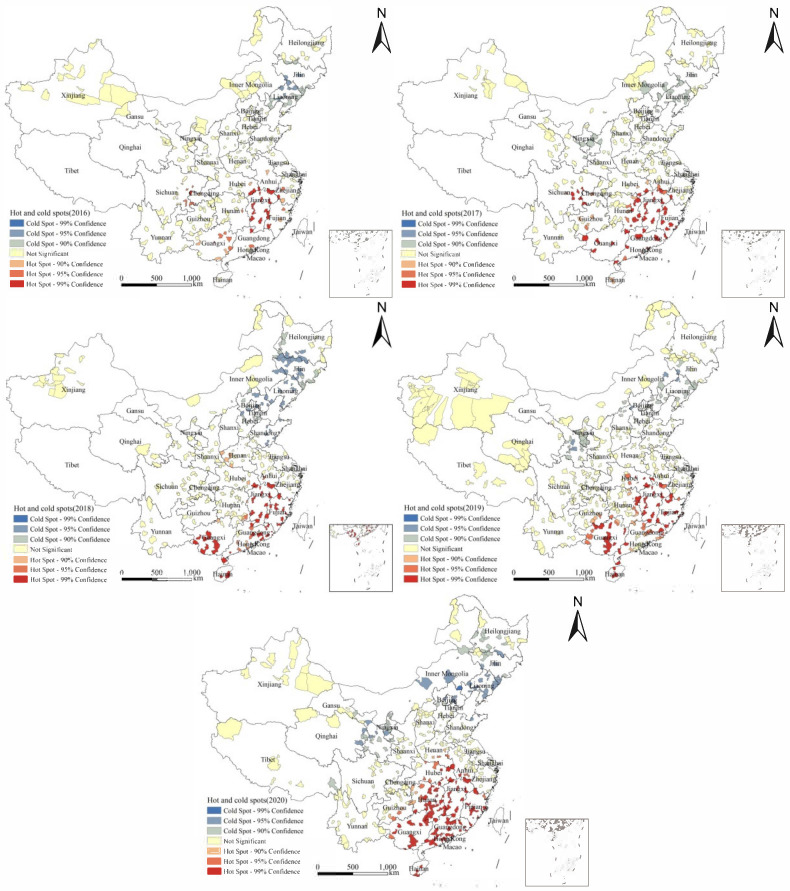
The county-level results of Getis-Ord Gi* from 2016 to 2020.

The results of the hotspot analysis at the county level (Figure 4) demonstrated that “hot spots” included partial districts and counties in Hainan, Guangxi, Guangdong, Fujian, Jiangxi, Hunan, Chongqing, Sichuan, Guizhou, Anhui, Hubei and Henan, while the “cold spots” included some districts and counties in Liaoning, Jilin, Heilongjiang, Inner Mongolia, Hebei, Ningxia and Gansu.

### 3.6. Spatio-Temporal Analysis

**Figure 5:**
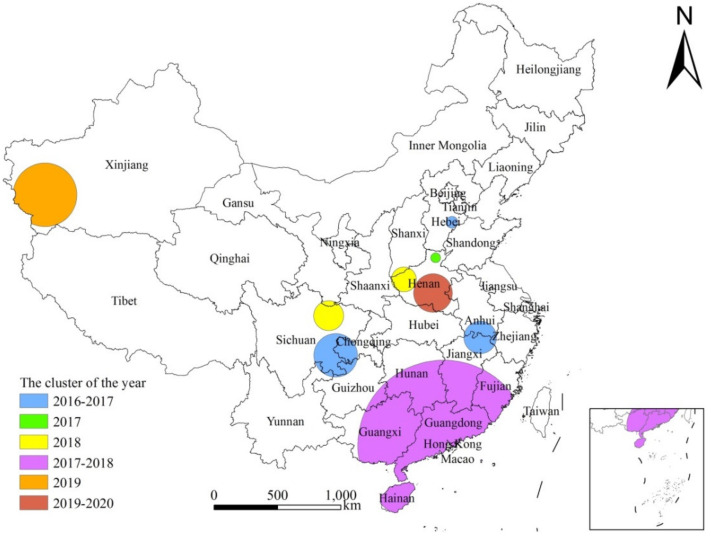
Space–time scanning analysis of *Enterobius vermicularis* infection among children in China from 2016 to 2020.

The cluster analysis of spatio-temporal scanning statistics at the county levels (Figure 5) illustrated that the infection rate of *E. vermicularis* among children in China showed 9 clustering areas from 2016 to 2020 (Table 5). The successive decrease in LLR indicated that the clustering intensity was weakening over the five years. From 2016 to 2018, there were 7 clustering areas, involving 317 counties belonging to 10 provinces, namely, Hainan, Guangxi, Guangdong, Fujian, Jiangxi, Hunan, Sichuan, Chongqing, Anhui and Hebei. The radius of the largest clustering area was 647.46 km. Two clustering areas were observed in 2019–2020, involving 43 counties belonging to Henan and Xinjiang. The radius of the largest clustering area here was 257.96 km.

## 4. Discussion

Enterobiasis is a common parasitic disease in children worldwide, especially in poor areas of developing countries. Due to a lack of attention, enterobiasis has affected the growth of children. In 2015, the national survey for major human parasitic diseases in China demonstrated that *E. vermicularis* infection was present in 28 provinces and children under 9 years were the majorly afflicted population [12]. From 2016 to 2020, China increased the control of soil-borne nematodiasis and established a surveillance network in which *E. vermicularis* infection in children aged 3 to 9 was included [26,27]. This supports the capture of the epidemic situation of enterobiasis in children in China, with benefit in formulating and improving intervention measures. 

The results of this study indicate that the infection rate of *E. vermicularis* in children aged 3 to 9 years in China dropped significantly, by 32% in 2020 compared to that in 2016. However, enterobiasis is still prevalent in some areas, where there exist a suitable climate, ecological environment, and production and lifestyle. The infection rate of boys and girls both decreased, while the difference was not significant in different genders. This result was consistent with the findings of the studies carried out in Shandong [28], Guangxi [29], Henan [30], Anhui, Fujian and other provinces [31]. In addition, the infection rate of *E. vermicularis* was the highest in children from the Zhuang ethnic group during 2016–2019, while it was the highest in children from Li in 2020, because they live in Guangxi and Hainan, where the climate and abundant rainfall are suitable for the survival and reproduction of the parasite. Additionally, poor hygiene habits, ethnic diets and poor living conditions coupled with a lack of awareness also cause a high infection rate [32,33,34]. In this study, the infection rate was higher in 4-, 5- and 6- year-old children, while a lower infection rate was observed in children aged 3 and 9. The high infection rate in children aged 4 to 6 years may be due to children in this age group starting to enter kindergarten, which increases the risk of infection [35]. The infection rate in the 3-year-old group was low, as these children were probably still at home or only attended kindergarten intermittently. Older children probably suffer less from *E. vermicularis* infection as personal hygiene habits improve with age and education level [36].

The results of the hotspot analysis at the provincial and county levels showed that the hotspots of *E. vermicularis* infection among children aged 3 to 9 in China were concentrated in the southern areas of the Yangtze River, such as Jiangxi, Guangxi and Hainan. The SaTScan spatial–temporal scanning analysis demonstrated that the largest clustering areas of infection among children included Hainan, Guangxi, Guangdong, Fujian and Jiangxi. From 2016 to 2020, the high infection areas were Jiangxi (14.29%), Guangxi (13.66%), Hainan (9.60%), Chongqing (9.40%), Guangdong (7.42%) and Fujian (7.20%) (Table 5), respectively. After comparison, it is consistent with the hotspot areas and the largest cluster areas. Firstly, the temperature and humidity in the southern region are higher compared to other regions, rendering the soil more suitable for the growth and development of *E. vermicularis*. Secondly, these areas are lacking in sanitation and hygiene, which favors infection [37,38]. 

## 5. Conclusions

In conclusion, *E. vermicularis* infection in children aged 3 to 9 years in China decreased considerably from 2016 to 2020 by 32% and the high infection rate was concentrated in 4-, 5- and 6-year-old children. However, there still exist high infection areas, mainly concentrated in the south of the Yangtze River in China. Thus, it is necessary to strengthen surveillance and implement control measures in combination with health education and environmental improvement. This study adds to the growing body of knowledge concerning the epidemic situation of enterobiasis among children aged 3 to 9 years in China, thus providing support for formulating prevention and control strategies and optimizing the allocation of resources for enterobiasis.

## Figures and Tables

**Table 1:** *Enterobius vermicularis* infection among children aged 3 to 9 years old in China from 2016 to 2020.

Province (Municipality/Autonomous Region)	2016 N (%) *	2017 N (%) *	2018 N (%) *	2019 N (%) *	2020 N (%) *	Total N (%) *
Beijing	0/161 (0.00)	0/77 (0.00)	0/449 (0.00)	3/374 (0.80)	0/322 (0.00)	3/1383 (0.22)
Tianjing	0/325 (0.00)	1/246 (0.41)	0/210 (0.00)	0/251 (0.00)	0/258 (0.00)	1/1290 (0.08)
Hebei	42/2821 (1.49)	22/2382 (0.92)	21/2197 (0.96)	28/2765 (1.01)	13/2454 (0.53)	126/12,619 (1.00)
Shanxi ^(1)^	10/1101 (0.91)	22/1336 (1.65)	/	6/2053 (0.29)	9/1821 (0.49)	47/6311 (0.74)
Inner Mongolia	0/768 (0.00)	2/790 (0.25)	0/1,392 (0.00)	1/1346 (0.07)	0/1225 (0.00)	3/5521 (0.05)
Liaoning	0/1153 (0.00)	0/1133 (0.00)	0/677 (0.00)	0/874 (0.00)	1/814 (0.12)	1/4651 (0.02)
Jilin	0/284 (0.00)	1/277 (0.36)	2/840 (0.24)	0/386 (0.00)	2/411 (0.49)	5/2198 (0.23)
Heilongjiang	0/1403 (0.00)	3/904 (0.33)	0/849 (0.00)	3/1251 (0.24)	0/646 (0.00)	6/5053 (0.12)
Shanghai	0/68 (0.00)	0/17 (0.00)	0/55 (0.00)	3/142 (2.11)	0/42 (0.00)	3/324 (0.93)
Jiangsu	0/214 (0.00)	4/1055 (0.38)	0/120 (0.00)	1/863 (0.12)	10/810 (1.23)	15/3062 (0.49)
Zhejiang	19/727 (2.61)	7/804 (0.87)	2/537 (0.37)	9/603 (1.49)	1/476 (0.21)	38/3147 (1.21)
Anhui	3/73 (4.11)	26/1849 (1.41)	16/1640 (0.98)	31/2229 (1.39)	24/2305 (1.04)	100/8096 (1.24)
Fujian	30/1094 (2.74)	113/2376 (4.76)	159/2208 (7.20)	159/2214 (7.18)	114/2449 (4.65)	575/10,341 (5.56)
Jiangxi	238/1964 (12.12)	256/1791 (14.29)	120/1409 (8.52)	110/1611 (6.83)	121/1550 (7.81)	845/8325 (10.15)
Shandong	27/1153 (2.34)	16/1526 (1.05)	8/1858 (0.43)	48/1941 (2.47)	21/1783 (1.18)	120/8261 (1.45)
Henan	137/4235 (3.23)	162/4846 (3.34)	176/4664 (3.77)	136/4596 (2.96)	132/3807 (3.47)	743/22,148 (3.35)
Hubei	11/1603 (0.69)	3/1389 (0.22)	37/2356 (1.57)	40/1758 (2.28)	31/1746 (1.78)	122/8852 (1.38)
Hunan	15/1598 (0.94)	113/4160 (2.72)	83/6598 (1.26)	120/5269 (2.28)	73/7070 (1.03)	404/24,695 (1.64)
Guangdong	50/913 (5.48)	174/2346 (7.42)	188/4122 (4.56)	178/5039 (3.53)	160/5743 (2.79)	750/18,163 (4.13)
Guangxi	139/2166 (6.42)	118/864 (13.66)	182/2232 (8.15)	238/3586 (6.64)	108/2285 (4.73)	785/11,133 (7.05)
Hainan	6/201 (2.99)	25/343 (7.29)	27/514 (5.25)	38/538 (7.06)	55/573 (9.60)	151/2169 (6.96)
Chongqing	13/527 (2.47)	42/447 (9.40)	3/560 (0.54)	1/478 (0.21)	4/732 (0.55)	63/2744 (2.30)
Sichuan	67/1293 (5.18)	49/1301 (3.77)	68/1421 (4.79)	77/3118 (2.47)	29/1194 (2.43)	290/8327 (3.48)
Guizhou	49/2028 (2.42)	33/2110 (1.56)	4/948 (0.42)	23/2699 (0.85)	29/2555 (1.14)	138/10,340 (1.33)
Yunnan	3/1336 (0.22)	6/1465 (0.41)	0/1016 (0.00)	11/2026 (0.54)	4/1834 (0.22)	24/7677 (0.31)
Tibet ^(2)^	/	/	/	1/1117 (0.09)	0/893 (0.00)	1/2010 (0.05)
Shaanxi	15/1228 (1.22)	0/1212 (0.00)	11/1365 (0.81)	15/1490 (1.01)	2/1167 (0.17)	43/6462 (0.67)
Gansu	7/1470 (0.48)	1/1414 (0.07)	1/1399 (0.07)	6/3164 (0.19)	6/3712 (0.16)	21/11,159 (0.19)
Qinghai	0/1044 (0.00)	0/615 (0.00)	0/893 (0.00)	0/1069 (0.00)	1/980 (0.10)	1/4601 (0.02)
Ningxia	21/853 (2.46)	1/435 (0.23)	3/665 (0.45)	21/2968 (0.71)	10/2338 (0.43)	56/7259 (0.77)
Xinjiang	9/2673 (0.34)	12/3119 (0.38)	22/2893 (0.76)	152/5774 (2.63)	8/3066 (0.26)	203/17,525 (1.16)

* N = No. infected/No. examined. (1) No surveillance was implemented in Shanxi Province in 2018. (2) No surveillances were implemented in Tibet from 2016 to 2018.

**Table 2:** *Enterobius vermicularis* infection among children by gender and ethnicity in China from 2016 to 2020.

Feature	2016 N (%) *	2017 N (%) *	2018 N (%) *	2019 N (%) *	2020 N (%) *	Total N (%) *
Gender						
Boys	479/19,497 (2.46)	650/22,647 (2.87)	592/24,601 (2.41)	781/33,666 (2.32)	513/30,008 (1.71)	3015/130,419 (2.31)
Girls	432/16,980 (2.54)	562/19,982 (2.81)	541/21,486 (2.52)	678/29,926 (2.27)	455/27,053 (1.68)	2668/115,427 (2.31)
Ethnicity						
Buyi	1/308 (0.32)	0/365 (0.00)	0/193 (0.00)	1/523 (0.19)	11/462 (2.38)	13/1851 (0.70)
Dong	8/128 (6.25)	6/419 (1.43)	7/196 (3.57)	0/96 (0.00)	7/633 (1.11)	28/1472 (1.90)
Han	764/28,455 (2.68)	1034/34,263 (3.02)	956/38,094 (2.51)	1091/47,492 (2.30)	863/45,098 (1.91)	4708/193,402 (2.43)
Hui	22/1039 (2.12)	2/997 (0.20)	5/656 (0.76)	13/2128 (0.61)	17/2,269 (0.75)	59/7089 (0.83)
Kazak	2/461 (0.43)	4/645 (0.62)	3/323 (0.93)	0/103 (0.00)	4/534 (0.75)	13/2066 (0.63)
Li	6/197 (3.05)	19/313 (6.07)	11/143 (7.69)	3/238 (1.26)	20/248 (8.06)	59/1139 (5.18)
Miao	19/709 (2.68)	12/937 (1.28)	10/330 (3.03)	6/574 (1.05)	5/924 (0.54)	52/3474 (1.50)
Tujia	2/833 (0.24)	3/681 (0.44)	2/684 (0.29)	0/516 (0.00)	1/832 (0.12)	8/3546 (0.23)
Uygur	3/1,644 (0.18)	2/963 (0.21)	17/1787 (0.95)	152/5020 (3.03)	1/1046 (0.10)	175/10,460 (1.67)
Yao	3/65 (4.62)	16/234 (6.84)	10/169 (5.92)	2/78 (2.56)	11/413 (2.66)	42/959 (4.38)
Zhuang	77/922 (8.35)	102/789 (12.93)	107/1111 (9.63)	174/1764 (9.86)	18/637 (2.83)	478/5223 (9.15)
Others	4/1716 (0.23)	12/2023 (0.59)	5/2401 (0.21)	17/5060 (0.34)	10/3965 (0.25)	48/15,165 (0.32)

* N = No. infected/No. examined.

**Table 3:** *Enterobius vermicularis* infection among children by ages in China from 2016 to 2020.

Ages	2016 N (%) *	2017 N (%) *	2018 N (%) *	2019 N (%) *	2020 N (%) *	Total N (%) *
3	117/4984 (2.35)	102/5655 (1.80)	122/6613 (1.84)	113/9262 (1.22)	81/8093 (1.00)	535/34,607 (1.55)
4	180/5505 (3.27)	199/6111 (3.26)	221/6868 (3.22)	213/8809 (2.42)	133/8551 (1.56)	946/35,844 (2.64)
5	144/5622 (2.56)	293/6917 (4.24)	210/7556 (2.78)	325/10,598 (3.07)	183/8582 (2.13)	1155/39,275 (2.94)
6	166/5484 (3.03)	226/6470 (3.49)	210/7262 (2.89)	294/9773 (3.01)	201/8921 (2.25)	1097/37,910 (2.89)
7	154/4971 (3.10)	154/5899 (2.61)	175/5953 (2.94)	208/8663 (2.40)	172/7764 (2.22)	863/33,250 (2.60)
8	91/5095 (1.79)	135/6108 (2.21)	118/5923 (1.99)	197/8580 (2.30)	127/8095 (1.57)	668/33,801 (1.98)
9	59/4816 (1.23)	103/5469 (1.88)	77/5912 (1.30)	109/7907 (1.38)	71/7055 (1.01)	419/31,159 (1.34)

* N = No. infected/No. examined.

**Table 4:** Global spatial autocorrelation analysis of *Enterobius vermicularis* infection among children in China from 2016 to 2020.

Year	Moran’s *I*	Expected Moran’s *I* value	Variance	Z value	*P* value
2016	0.154787	−0.034483	0.004674	2.768400	0.005633
2017	0.235557	−0.034483	0.00531	3.705713	0.000211
2018	0.226277	−0.035714	0.006286	3.304426	0.000952
2019	0.314987	−0.033333	0.008858	3.700856	0.000215
2020	0.230413	−0.033333	0.007734	2.999004	0.002709

**Table 5:** Cluster analysis of *Enterobius vermicularis* infection among children in China from 2016 to 2020 by spatial and space–time scan statistic.

Year	Cluster Center (°)		Radius (km)	No. of Clustered Counties	LLR	No. of Observed	No. of Expected	*P* Value
	Latitude	Longitude						
2017–2018	22.5199	113.3940	647.46	234	532.57	1359	523.50	0.0000
2016–2017	29.5538	117.2960	128.07	11	238.40	184	21.04	0.0000
2016–2017	29.1221	105.4950	171.33	37	110.50	150	33.24	0.0000
2018	34.3418	111.4940	99.94	13	65.64	80	15.87	0.0000
2019	37.2055	78.5104	257.96	10	53.63	124	41.60	0.0000
2019–2020	33.1618	113.8890	154.52	33	39.56	177	84.13	0.0000
2018	31.9484	104.9240	118.98	15	27.97	34	6.70	0.0000
2016–2017	38.0833	116.3760	47.61	3	18.12	51	19.19	0.0001
2017	35.6715	114.4770	39.42	4	12.54	32	11.36	0.0190

° A unit of measure for latitude and longitude, the combination of latitude and longitude can mark any location on Earth.

## Data Availability

Data cannot be shared publicly because of personal information presented in the database. Data are deposited in the National Institute of Parasitic Diseases, Chinese Center for Disease Control and Prevention (Chinese Center for Tropical Diseases Research), of which access is available through corresponding author (lisz@chinacdc.cn) for researchers who meet the criteria for access to confidential data.
